# Food insecurity and common mental disorders in perinatal women living in low socio-economic settings in Cape Town, South Africa during the COVID-19 pandemic: a cohort study

**DOI:** 10.1017/gmh.2022.12

**Published:** 2022-02-04

**Authors:** Zulfa Abrahams, Crick Lund

**Affiliations:** 1 Department of Psychiatry and Mental Health, Alan J Flisher Centre for Public Mental Health, University of Cape Town, Cape Town, South Africa; 2 Health Service and Population Research Department, Centre for Global Mental Health, Institute of Psychiatry, Psychology and Neuroscience, King's Global Health Institute, King's College London, London, UK

**Keywords:** COVID, depression, domestic violence, food security, perinatal

## Abstract

**Background:**

Common mental disorders (CMDs), i.e. depression and anxiety, are highly prevalent during the perinatal period, and is associated with poverty, food insecurity and domestic violence. We collected data from perinatal women at two time-points during the COVID-19 pandemic to test the hypotheses that (1) socio-economic adversities at baseline would be associated with CMD prevalence at follow-up and (2) worse mental health at baseline would be associated with higher food insecurity prevalence at follow-up.

**Methods:**

Telephonic interviews with perinatal women attending healthcare facilities in Cape Town, South Africa. Multivariable (multilevel) regression analysis was used to model the associations of baseline risk factors with the prevalence of household food insecurity and probable CMD at 3 months follow-up.

**Results:**

At baseline 859 women were recruited, of whom 217 (25%) were pregnant, 631 (73%) had given birth in the previous 6 months, 106 (12%) had probable CMD, and 375 (44%) were severely food insecure. At follow-up (*n* = 634), 22 (4%) were still pregnant, 603 (95%) had given birth, 44 (7%) had probable CMD, and 207 (33%) were severely food insecure. In the multivariable regression model, after controlling for confounders, unemployment [incidence rate ratio (IRR) 1.19 (1.12–2.27); *p* < 0.001] and had higher scores on the Edinburgh Postnatal Depression Scale [IRR 1.05 (1.03–1.09); *p* < 0.001] at baseline predicted food insecurity at follow-up; and experiencing domestic violence [OR 2.79 (1.41–5.50); *p* = 0.003] at baseline predicted CMD at follow-up.

**Conclusions:**

This study highlights the complex bidirectional relationship between mental health and socio-economic adversity among perinatal women during the COVID-19 pandemic.

## Background

In low- and middle-income countries, common mental disorders (CMDs) such as depression and anxiety disorders are highly prevalent during the perinatal period (van Heyningen *et al*., 2016, 2017; Redinger *et al*., 2018) and are associated with several markers of multidimensional poverty, food insecurity (Jebena *et al*., 2015; Abrahams *et al*., 2018) and domestic violence (Malan *et al*., 2018). The prevalence appears to fluctuate during pregnancy – with women in the first and third trimester of pregnancy, and those with other children, being at greater risk of depression and anxiety (Yan *et al*., 2020). Moreover, pregnant women with mild to moderate symptoms of depression during their first and second trimester of pregnancy show a natural remission of symptoms during the perinatal period, even without intervention (Lee *et al*., 2014; Garman *et al*., 2019).

South Africa is characterised by high levels of inequality and multidimensional poverty (Fransman and Yu, 2019; Posel and Rogan, 2019), evidenced by having one of the highest Gini coefficients in the world (The World Bank, 2021). Reflecting this high inequality, South Africa is food secure at a national level, i.e. enough food is being produced and imported to ensure that all South Africans have sufficient food, yet more than 20% of South African households are food insecure (Statistics South Africa, 2019*a*). Household food insecurity exists when households do not have ‘physical, social and economic access to sufficient, safe and nutritious food that meets their dietary needs and food preferences for an active and healthy life’ (Food and Agriculture Organisation of the United States, 2001). Based on the 2017 General Household Survey in South Africa, households with the greatest levels of food insecurity are headed by Black Africans, have more than eight household members and are living in urban areas (Statistics South Africa, 2019*a*).

Late in March 2020, the South African government enforced a national lockdown to curb the spread of the Coronavirus (COVID-19) (South African Government, 2020*d*). For more than 2 months, the majority of South Africans were confined to their homes, except those performing essential services (South African Government, 2020*b*, 2020*c*). Even when restrictions started easing in June 2020, many industries remained under lockdown (South African Government, 2020*a*). The COVID-19 pandemic triggered an increase in unemployment and poverty in South Africa, affecting already vulnerable populations, especially those working in the informal sector who were unskilled and had low levels of education (Rogan and Skinner, 2020). Unemployment rates, which had remained at about 20% for the last decade, rose to 31% by September 2020 (Statistics South Africa, 2021). As a result of the high levels of unemployment, raised food prices and economic decline (Pereira and Oliveira, 2020), acute food insecurity was experienced throughout South Africa (Integrated Food Security Phase Classification, 2021). Food security is especially important for pregnant and lactating women as inadequate nutrition during pregnancy, lactation and infancy is associated with poor infant and child health outcomes (Koletzko *et al*., 2019).

The relationship between CMDs and food insecurity is thought to be bidirectional, including during the perinatal period (Huddleston-Casas *et al*., 2009). The social causation hypothesis proposes that those living in poverty and food insecurity develop poor mental health because of exposure to a number of adversities, including stress resulting from their economic difficulties, while the social drift hypothesis proposes that poor mental health causes disability and associated loss of employment together with increased medical expenditure, resulting in a drift into poverty (Lund and Cois, 2018; Ridley *et al*., 2020). While many studies have reported cross-sectional associations between poverty and CMDs in perinatal women, few studies have used longitudinal designs to examine the bidirectional relationship between mental health and socio-economic risk factors (Lund *et al*., 2018). To our knowledge, no longitudinal studies have reported on the relationship between household food insecurity and CMDs in perinatal women living in South Africa during the COVID-19 pandemic. We hypothesised that socio-economic adversities at baseline would predict higher CMD prevalence at follow-up and in turn that worse mental health experienced at baseline would predict higher food insecurity prevalence at follow-up. To test these hypotheses, we used data collected at two time-points from perinatal women living in low socio-economic settings in Cape Town during the COVID-19 pandemic, to explore the association of several baseline risk factors with the prevalence of household food insecurity and probable CMD experienced at follow-up.

## Methods

### Setting

This analysis was conducted as part of the Health Systems Strengthening in sub-Saharan Africa (ASSET) study (ASSET, 2020) which included a planned cluster randomised control trial (ISRTCN41483663) to evaluate an intervention to strengthen detection, referral and care for antenatal women with probable CMDs and experiences of domestic violence in Cape Town, South Africa. Study sites consisted of 14 midwife obstetric units (MOUs) or basic antenatal care (BANC) clinics that were randomly selected from 23 facilities in the Cape Metropolitan Health District in Cape Town. As part of the study, the clinical notes of 2149 perinatal women were reviewed in February and March 2020. Patients' contact details, and gestational and medical history were captured during the review.

All healthcare facilities are managed by the Western Cape Department of Health and offer free antenatal and postnatal care. All facilities are situated in low-resource urban communities, across a large flat area known as the ‘Cape Flats’ (South African History Online, 2020). Black and Coloured [people of mixed ancestry (Adhikari, 2005)] families were forcibly moved to these areas during the Apartheid era in South Africa, and now reside in low-cost housing and informal settlements, where residents experience high levels of unemployment and poverty (Statistics South Africa, 2017), gang violence (Maringira and Masiya, 2018) and domestic violence (Malan *et al*., 2018). During the pandemic, a survey among perinatal women living in vulnerable communities in Cape Town reported that 71% of women were unemployed, and almost 40% of women reported that they had gone to bed hungry due to lack of food in the household (Matlwa Mabaso *et al*., 2021).

### Data collection

**Fig. 1.**
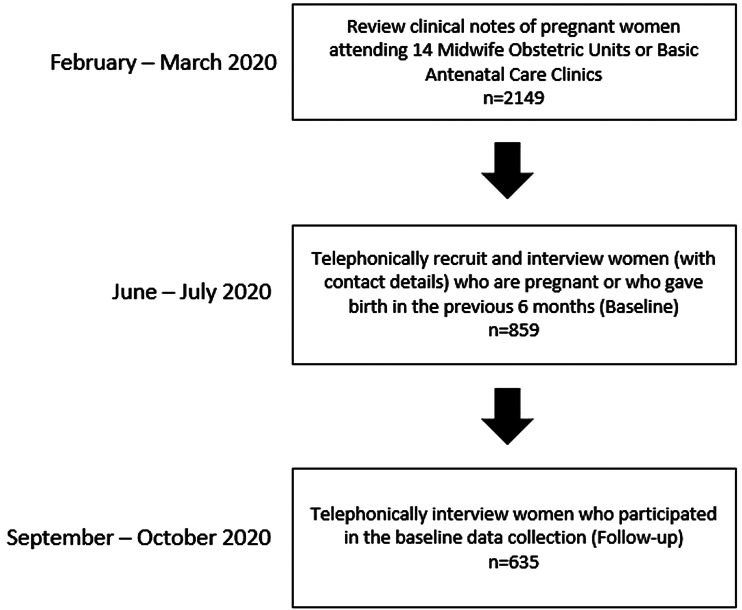
Participant selection flowchart.

Between June and July 2020 (baseline), using the contact details obtained from 2149 pregnant women's clinical notes (Fig. 1), perinatal women were telephonically contacted and invited to participate in a telephonic survey. Trained fieldworkers verbally explained the study in participants' home language (which included explaining the sensitive and confidential nature of the questions) before seeking informed consent. The following questionnaires were administered to women who consented to participate in the study: (1) a socio-demographic questionnaire, (2) the Edinburgh Postnatal Depression Scale (EPDS) (Cox *et al*., 1987), (3) a three-question mental health screening questionnaire (Abrahams *et al*., 2019), (4) the Household Food Insecurity and Access Scale (HFIAS) (Castell *et al*., 2015), and (5) the short form of the Composite Abuse Scale (CAS-SF) (Ford-Gilboe *et al*., 2016). Three months later (follow-up), the same questionnaires were telephonically administered to women who participated in the baseline data collection.

*Probable CMD* was measured using the EPDS, a 10-question screening tool with a 7-day recall period. It has been validated against the Diagnostic and Statistical Manual (DSM-IV) (Frances *et al*., 1989; Sheehan *et al*., 1998) for depression and anxiety in a sample of postnatal women in South Africa (Cox *et al*., 1987; Lawrie *et al*., 1998) with a cut-off of ⩾13 indicating a probable CMD (sensitivity = 80%; specificity = 76.6%) (Cox *et al*., 1987).

*Psychological distress* was measured using a three-question mental health screening questionnaire with a 14-day recall period. This questionnaire has been validated against the EPDS with a cut-point of ⩾2 indicating the presence of psychological distress (sensitivity = 85.7%; specificity = 92.9%) (Abrahams *et al*., 2019).

*Household food insecurity* was measured with the HFIAS, a nine-item scale with a 30-day recall period which measures the household's frequency of running out of food or eating inadequate amounts of food (Castell *et al*., 2015). The HFIAS was used to develop a categorical variable to describe four levels of household food insecurity experienced during the past 30 days: food secure, mildly food insecure, moderately food insecure and severely food insecure (Coates *et al*., 2007), as well as a binary variable which differentiates between food secure and food insecure households. Food insecure refers to households that experienced mild, moderate or severe food insecurity.

*Domestic violence* was measured with the 15-item CAS-SF (Ford-Gilboe *et al*., 2016), which captures self-reported experiences and the frequency of psychological, physical and sexual abuse. At baseline the recall period was 12 months, while at follow-up women were asked whether they had experienced any form of domestic violence since the previous interview, approximately 3 months earlier.

### Data analysis

Data analysis was carried out using STATA/SE statistical software package version 15.1 (StataCorp., College Station, TX, USA). Participants with more than 30% incomplete data were excluded from the analysis. Multiple imputation was used to account for data assumed to be missing at random. Categorical variables were described using frequency and percentages, and associations measured using χ^2^ tests.

We tested the hypotheses that socio-economic adversities at baseline would predict higher CMD prevalence at follow-up and in turn that worse mental health experienced at baseline would predict higher food insecurity prevalence at follow-up. Using multilevel linear regression, we modelled the associations of several baseline risk factors with the prevalence of food insecurity and domestic violence at follow-up. Using multilevel logistic regression, we modelled the associations of several baseline risk factors with the prevalence of probable CMD at follow-up. We used baseline food insecurity, baseline CAS-SF scores and baseline EPDS scores to account for the hierarchical structure of the data. Univariate analysis was used to explore the unadjusted association between covariates and household food insecurity and probable CMD outcomes. A purposeful selection model building strategy (Zhang, 2016) was used to build the multivariable model. Starting with an empty model, variables with a *p* value of <0.25 (Bursac *et al*., 2008) in the univariate model were added. Partial likelihood ratio tests were used to compare the models.

### Ethical approval

Ethical approval for the study was obtained from the Human Research Ethics Committee at the Faculty of Health Sciences, University of Cape Town (Ref No: 139/2018) and the Psychiatry, Nursing and Midwifery Research Ethics Subcommittee at Kings College London (Ref No: 17/18-7807). The Western Cape Department of Health approved the use of the research sites (Ref No: WC_201807_008). Consent forms were available in English, Afrikaans and isiXhosa. All participants were informed that they were free to withdraw from the study at any time without consequences. Women who screened positive for probable CMDs or experiences of domestic violence or severe food insecurity were provided with a resource list of organisations that provided telephonic counselling and support. No financial incentives were provided for participating in the study.

## Results

At baseline, 859 perinatal women were recruited and participated in telephonic interviews. Three months later, 635 (74%) women participated in the follow-up telephonic interviews (Fig. 1). The women who were lost to follow-up were no longer contactable on the cell phone number used at baseline. Those lost to follow-up were not significantly different to those who participated in the follow-up interviews with regards to age (*p* = 0.557), employment status (*p* = 0.059), number of children (0.557), receiving financial support from their partner (*p* = 0.144) and number of people per sleeping room (*p* = 0.618). The prevalence of probable CMD in those who were pregnant was not significantly different to those who were not pregnant (18% *v*. 11%; *p* = 0.085).

At follow-up (Table 1), significantly more women were unemployed (71% *v.* 54%; *p* < 0.001) and had given birth in the previous 6 months (95% *v.* 74%; *p* < 0.001), compared to baseline. Significantly fewer women experienced psychological distress (14% *v.* 26%; *p* < 0.001) and had a probable CMD (7% *v.* 12%; *p* < 0.001) at follow-up, compared to baseline. At follow-up, the prevalence of food insecurity had significantly reduced (*p* < 0.001). More women were food secure (31% *v.* 19%) and mildly food insecure (13% *v.* 9%), while fewer women were moderately (24% *v.* 28%) and severely food insecure (33% *v.* 44%) at follow-up, compared to baseline. Experiences of any form of domestic violence decreased significantly (12% *v.* 21%; *p* < 0.001) from baseline to follow-up.

**Table 1.** Socio-demographic and clinical characteristics of participants at baseline and follow-up

	Baseline (*n* = 859) *n* (%)	Follow-up (*n* = 635) *n* (%)	*p* value^a^
Age			
<18 years	46 (5.4)	37 (5.8)	0.959
18–24 years	260 (30.3)	186 (29.3)	
25–35 years	464 (54.0)	344 (54.2)	
>35 years	89 (10.4)	68 (10.7)	
Race			
Black African	609 (70.9)	476 (75.0)	0.082
Mixed ancestry	250 (29.1)	159 (25.0)	
Other children (from previous pregnancies)			
No children	325 (37.8)	240 (37.8)	0.925
1–2 children	442 (51.5)	323 (50.9)	
⩾3 children	92 (10.7)	72 (11.3)	
Planned pregnancy	309 (36.0)	231 (36.4)	0.872
Employment status			
Employed	338 (39.4)	151 (23.8)	**<0.001**
Unemployed	466 (54.3)	450 (70.9)	
Student	55 (6.4)	34 (5.4)	
Financial support from partner	346 (40.3)	264 (41.7)	0.572
Any social support grant	454 (52.9)	334 (52.6)	0.923
Child support grant	41 448.2	29 947.1	0.671
People per sleeping room			
1–2 people	420 (48.9)	310 (48.8)	0.825
3–4 people	372 (43.3)	270 (42.5)	
⩾5 people	67 (7.8)	55 (8.7)	
HIV-infected	156 (18.2)	126 (19.8)	0.412
Pregnancy status			
Pregnant	217 (25.3)	22 (3.5)	**<0.001**
⩽6 months after birth	631 (73.5)	603 (95.0)	
Miscarriage/stillbirth	11 (1.3)	10 (1.6)	
Psychological distress^b^	226 (26.3)	87 (13.7)	**<0.001**
Probable CMD^c^	106 (12.3)	46 (7.2)	**0.001**
EPDS score (mean ± s.d.)	5.72 ± 5.33	4.33 ± 4.63	**<0.001**^d^
Household food insecurity access prevalence (HFIAP)			
Food secure	165 (19.2)	195 (30.7)	**<0.001**
Mildly food insecure	80 (9.3)	81(12.8)	
Moderately food insecure	239 (27.8)	152 (23.9)	
Severely food insecure	375 (43.7)	207 (32.6)	
Domestic violence			
Psychological abuse	138 (16.1)	60 (9.5)	**<0.001**
Physical abuse	121 (14.1)	54 (8.5)	**0.001**
Sexual abuse	14 (1.6)	5 (0.8)	0.151
Any abuse	179 (20.8)	78 (12.3)	**<0.001**

s.d., standard deviation.

^a^*χ*^2^ test.

^b^⩾2 on the three-question mental health screening questionnaire.

^c^Common mental disorder, ⩾13 on the Edinburgh Postnatal Depression Scale (EPDS).

^d^*t*-test.

In the cross-sectional analysis, at baseline, several markers of multidimensional poverty and worse mental health were significantly higher in women who were food insecure compared to those who were food secure. These markers included being unemployed (58% *v.* 39%; *p* < 0.001), receiving a child support grant (50% *v.* 39%; *p* = 0.012), having ⩾5 people per sleeping room (8.5% *v.* 5%; *p* = 0.005), experiencing psychological distress (30% *v.* 13%; *p* < 0.001), having a probable CMD (14% *v.* 4%; *p* < 0.001) and experiencing physical abuse (15% *v.* 9%; *p* < 0.001) (Table 2). At follow-up, the pattern remained the same – significantly more women who were food insecure were unemployed (56% *v.* 40%; *p* < 0.001), received a child support grant (51% *v.* 38%; *p* = 0.002), experienced psychological distress (16% *v.* 8%; *p* = 0.003), had a probable CMD (9% *v.* 4%; *p* = 0.042) and experienced physical abuse (11% *v.* 4%; *p* = 0.003), compared to those who were food secure. In addition, at follow-up, significantly fewer women who were food insecure had planned their pregnancy (34% *v.* 43%; *p* = 0.031), while significantly more women who were food insecure were HIV-infected (23% *v.* 12%; *p* = 0.002), compared to those who were food secure.

**Table 2.** Bivariate associations with food security prevalence at baseline and follow-up

	Baseline			Follow-up		
	Food secure *n* (%) 165 (19.2)	Food insecure^a^ *n* (%) 694 (80.8)	*p* value^b^	Food secure *n* (%) 195 (30.7)	Food insecure *n* (%) 440 (69.3)	*p* value^b^
Age						
<18 years	12 (7.3)	34 (4.9)	0.434	11 (5.6)	26 (5.9)	0.060
18–24 years	52 (31.5)	208 (30.0)		68 (34.9)	118 (26.8)	
25–35 years	88 (53.3)	376 (54.2)		103 (52.8)	241 (54.8)	
>35 years	13 (7.9)	76 (10.9)		13 (6.7)	55 (12.5)	
Race						
Black	104 (72.8)	505 (63.0)	**0.013**	118 (60.5)	358 (81.4)	**<0.001**
Mixed ancestry	61 (27.2)	189 (37.0)		77 (39.5)	82 (18.6)	
Other children (from previous pregnancies)						
No children	78 (47.3)	247 (35.6)	**0.005**	87 (44.6)	153 (34.8)	**0.010**
1–2 children	78 (47.3)	364 (52.4)		95 (48.7)	228 (51.8)	
⩾3 children	9 (5.4)	83 (12.0)		13 (6.7)	59 (13.4)	
Planned pregnancy	67 (21.7)	242 (78.3)	0.168	83 (42.6)	148 (33.6)	**0.031**
Employment status						
Employed	82 (49.7)	256 (36.9)	**<0.001**	111 (56.9)	166 (37.7)	**<0.001**
Unemployed	64 (38.8)	402 (57.9)		79 (40.5)	248 (56.4)	
Student	19 (11.5)	36 (5.2)		5 (2.6)	26 (5.9)	
Financial support from partner	78 (47.3)	268 (38.6)	**0.042**	96 (49.2)	16 (38.4)	**0.011**
Any social support grant	75 (54.5)	379 (45.4)	**0.034**	78 (40.0)	232 (52.7)	**0.003**
Child support grant	65 (39.4)	349 (50.3)	**0.012**	74 (37.9)	225 (51.1)	**0.002**
People per sleeping room						
1–2 people	99 (60.0)	321 (46.3)	**0.005**	107 (54.9)	203 (46.1)	**0.083**
3–4 people	58 (35.2)	314 (45.2)		76 (39.0)	194 (44.1)	
⩾5 people	8 (4.9)	59 (8.5)		12 (6.2)	43 (9.8)	
HIV-infected	23 (13.9)	133 (19.2)	0.118	23 (11.8)	103 (23.4)	**0.001**
Pregnancy status						
Pregnant	60 (36.4)	157 (22.6)	**0.001**	12 (6.2)	10 (2.3)	**0.037**
⩽6 months after birth	103 (62.4)	528 (76.1)		179 (91.8)	424 (96.4)	
Miscarriage/stillbirth	2 (1.2)	9 (1.3)		4 (2.0)	6 (1.4)	
Psychological distress^c^	21 (12.7)	205 (29.5)	**<0.001**	15 (7.7)	72 (16.4)	**0.003**
Probable CMD^d^	7 (4.2)	99 (14.3)	**<0.001**	8 (4.1)	38 (8.6)	**0.042**
Domestic violence						
Psychological abuse	19 (11.5)	119 (17.2)	0.077	14 (7.2)	46 (10.4)	0.193
Physical abuse	15 (9.1)	106 (15.3)	**0.040**	7 (3.6)	47 (10.7)	**0.003**
Sexual abuse	0	14 (2.0)	0.066	0	5 (1.1)	0.135
Any abuse	24 (14.5)	155 (22.3)	**0.027**	15 (7.7)	63 (14.3)	**0.019**

^a^Food insecure (mild, moderate and severely food insecure).

^b^*χ*^2^ test.

^c^ ⩾ 2 on the psychological distress questionnaire.

^d^Common mental disorder, ⩾13 on the Edinburgh Postnatal Depression Scale (EPDS).

In the cross-sectional analysis, at baseline, significantly more women with probable CMD were of mixed ancestry (45% *v.* 27%; *p* < 0.001), were still pregnant (32% *v.* 24%; *p* = 0.009), experienced psychological distress (76% *v.* 19%; *p* < 0.001), were food insecure (93% *v.* 79%; *p* < 0.001) and experienced domestic violence (42% *v.* 18%; *p* < 0.001) compared to those without probable CMD (Table 3). At follow-up, significantly more women with probable CMD experienced psychological distress (67% *v.* 10%; *p* < 0.001), were food insecure (83% *v.* 68%; *p* = 0.042) and experienced psychological abuse (30% *v.* 8%; *p* < 0.001), physical abuse (17% *v.* 8%; *p* = 0.025) or any form of abuse (30% *v.* 10%; *p* < 0.001), compared to those without probable CMD.

**Table 3.** Bivariate cross-sectional associations with common mental disorder (CMD) at baseline and follow-up

	Baseline			Follow-up		
	With CMD^a^ *n* (%) 106 (12.3)	Without CMD *n* (%) 753 (87.7)	*p* value^b^	With CMD *n* (%) 46 (7.2)	Without CMD *n* (%) 589 (92.8)	*p* value^b^
Age						
<18 years	7 (6.6)	39 (5.2)	0.231	3 (6.5)	34 (5.8)	0.083
18–24 years	23 (21.7)	237 (31.5)		6 (13.0)	180 (30.6)	
25–35 years	64 (60.4)	400 (53.1)		32 (69.6)	312 (53.0)	
>35 years	12 (11.3)	77 (10.2)		5 (10.9)	63 (10.7)	
Race						
Black	58 (54.7)	551 (73.2)	**<0.001**	32 (72.7)	413 (73.9)	0.867
Mixed ancestry	48 (45.3)	202 (26.8)		12 (27.3)	146 (26.1)	
Other children (from previous pregnancies)						
No children	35 (33.0)	290 (38.5)	0.456	16 (34.8)	224 (38.0)	0.199
1–2 children	57 (53.8)	385 (51.1)		28 (60.9)	295 (50.1)	
⩾3 children	14 (13.2)	78 (10.4)		2 (4.4)	70 (11.9)	
Planned pregnancy	33 (31.1)	276 (36.7)	0.267	18 (39.1)	231 (36.2)	0.687
Employment status						
Employed	41 (38.7)	297 (39.4)	0.875	15 (32.6)	232 (39.4)	0.579
Unemployed	57 (53.8)	409 (54.3)		27 (58.7)	321 (54.5)	
Student	8 (7.6)	47 (6.2)		4 (8.7)	36 (6.1)	
Financial support from partner	41 (38.7)	305 (40.5)	0.720	16 (34.8)	249 (42.3)	0.321
Any social support grant	57 (53.8)	371 (49.3)	0.385	25 (54.4)	288 (48.9)	0.476
Child support grant	56 (52.8)	358 (47.5)	0.308	25 (54.4)	279 (47.4)	0.361
People per sleeping room						
1–2 people	56 (52.8)	364 (48.3)	0.665	23 (50.0)	287 (48.7)	0.478
3–4 people	43 (40.6)	329 (43.7)		17 (37.0)	253 (43.0)	
⩾5 people	7 (6.6)	60 (8.0)		46 (13.0)	49 (8.3)	
HIV-infected	20 (18.9)	136 (18.1)	0.840	11 (23.9)	1158 (19.5)	0.472
Pregnancy status						
Pregnant	34 (32.1)	183 (24.3)	**0.009**	2 (4.4)	20 (3.4)	0.272
⩽6 months after birth	68 (64.2)	563 (74.8)		42 (91.3)	561 (95.3)	
Miscarriage/stillbirth	4 (3.8)	7 (0.9)		2 (4.4)	8 (1.4)	
Psychological distress^c^	80 (75.5)	146 (19.4)	**<0.001**	31 (67.4)	56 (9.5)	**<0.001**
Food insecure	99 (93.4)	595 (79.02)	**<0.001**	38 (82.6)	402 (68.2)	**0.042**
Domestic violence						
Psychological abuse	37 (34.9)	101 (13.4)	**<0.001**	14 (30.4)	46 (7.8)	**<0.001**
Physical abuse	33 (31.1)	88 (11.7)	**<0.001**	8 (17.4)	46 (7.8)	**0.025**
Sexual abuse	8 (7.6)	6 (0.8)	**<0.001**	1 (2.2)	4 (0.7)	0.269
Any abuse	44 (41.5)	135 (17.9)	**<0.001**	14 (30.4)	64 (10.9)	**<0.001**

^a^⩾13 on the Edinburgh Postnatal Depression Scale (EPDS).

^b^*χ*^2^ test.

^c^⩾2 on the psychological distress questionnaire.

In the multivariable linear regression model with HFIAS scores as the outcome variable (Table 4), after controlling for confounders (age, ethnicity, number of children, having a planned pregnancy, receiving a social support grant and being HIV-infected), the risk of higher HFIAS scores, i.e. being food insecure, was greater in women who were unemployed [incidence rate ratio (IRR) 1.19 (95% CI 1.12–1.27); *p* < 0.001] and with higher EPDS scores [IRR 1.02 (95% CI 1.01–1.03); *p* < 0.001] at baseline. In the multivariable logistic regression model with probable CMD as the outcome variable (Table 5), after controlling for age, and number of children, the odds of experiencing probable CMD were greater in women who experienced domestic violence [OR 2.79 (95% CI 1.41–5.50); *p* = 0.003] at baseline. In the multivariable linear regression model with CAS-SF scores as the outcome variable (Table 6), after controlling for ethnicity and age, the risk of higher CAS-SF scores, i.e. domestic violence, was higher in women with probable CMD at baseline [IRR 1.35 (95% CI 1.02–1.79); *p* = 0.038].

**Table 4.** Multilevel linear regression model: baseline characteristics associated with the household food insecurity on the HFIAS

	Unadjusted model		Multivariable model	
	Incidence rate ratio (95% CI)	*p* value	Incidence rate ratio (95% CI)	*p* value
18–24 years [ref: <18 years]	0.67 (0.46–0.99)	0.042		
25–35 years [ref: <18 years]	1.08 (0.75–1.54)	0.680		
>35 years [ref: <18 years]	1.88 (0.97–3.64)	0.060		
Black [ref: Coloured]	2.50 (1.68–3.71)	<0.001	1.26 (1.16–1.36)	<0.001
1–2 child [ref: no children]	1.05 (0.73–1.50)	0.785		
⩾3 children [ref: no children]	2.14 (1.09–4.19)	0.026		
Planned pregnancy	0.68 (0.47–0.98)	0.042		
Unemployed [ref: employed]	1.44 (1.00–2.07)	0.048	1.19 (1.12–2.27)	<0.001
Student [ref: employed]	2.15 (0.94–4.91)	0.068		
Financial support from partner	0.67 (0.46–0.95)	0.027		
Child support grant	1.56 (1.08–2.24)	0.004		
Any social support grant	1.62 (1.13–2.33)	0.009	1.11 (1.04–1.18)	0.001
People/sleeping room	1.11 (0.95–1.29)	0.191		
HIV-infected	2.02 (1.22–3.37)	0.007	1.10 (1.04–1.18)	0.014
⩽6 months post birth [ref: pregnant]	1.33 (0.90–1.96)	0.156		
Miscarriage/stillbirth [ref: pregnant]	0.64 (0.17–2.47)	0.519		
Edinburgh Postnatal Depression Scale (EPDS) score	1.03 (1.00–1.07)	0.051	1.05 (1.01–1.03)	<0.001
Psychological abuse	1.11 (0.68–1.83)	0.674		
Physical abuse	0.91 (0.54–1.54)	0.725		
Sexual abuse	1.08 (0.22–5.26)	0.928		
Any abuse	1.04 (0.66–1.63)	0.872

**Table 5.** Multilevel logistic regression model: baseline characteristics associated with the prevalence of CMD at follow-up

	Unadjusted model		Multivariable model	
	Odds ratio (95% CI)	*p* value	Odds ratio (95% CI)	*p* value
18–24 years [ref: <18 years]	0.36 (0.14–0.87)	0.023	0.30 (0.12–0.74)	0.009
25–35 years [ref: <18 years]	2.05 (1.05–3.98)	0.035		
>35 years [ref: <18 years]	0.97 (0.36–2.64)	0.958		
Black [ref: Coloured]	1.22 (0.58–2.55)	0.602		
1–2 child [ref: no children]	1.44 (0.76–2.72)	0.259		
⩾3 children [ref: no children]	0.32 (0.07–1.37)	0.124	0.23 (0.05–1.02)	0.053
Planned pregnancy	1.26 (0.66–2.42)	0.470		
Unemployed [ref: employed]	1.08 (0.56–2.03)	0.808		
Student [ref: employed]	1.53 (0.50–4.73)	0.459		
Financial support from partner	0.76 (0.40–1.46)	0.410		
Child support grant	1.22 (0.65–2.27)	0.539		
Any social support grant	1.14 (0.61–2.13)	0.676		
People/sleeping room	1.03 (0.81–1.31)	0.827		
HIV-infected	1.31 (0.63–2.74)	0.469		
⩽6 months after birth [ref: pregnant]	1.37 (0.66–2.87)	0.403		
Miscarriage/stillbirth [ref: pregnant]	3.38 (0.57–19.89)	0.178		
Food insecure	2.02 (0.93–4.41)	0.077		
Psychological abuse	2.27 (1.10–4.68)	0.026		
Physical abuse	2.08 (0.97–4.43)	0.058		
Sexual abuse	4.77 (0.96–23.66)	0.056		
Any abuse	2.61 (1.33–5.13)	0.005	2.79 (1.41–5.50)	0.003

**Table 6.** Multilevel linear regression model: baseline characteristics associated with the prevalence of domestic violence on the CAS-SF

	Unadjusted model		Multivariable model	
	Incidence rate ratio (95% CI)	*p* value	Incidence rate ratio (95% CI)	*p* value
18–24 years [ref: <18 years]	0.84 (0.50–1.44)	0.540		
25–35 years [ref: <18 years]	1.34 (0.84–2.15)	0.215		
>35 years [ref: <18 years]	0.40 (0.14–1.12)	0.081	0.29 (0.16–0.51)	<0.001
Black [ref: Coloured]	0.63 (0.40–1.01)	0.056	0.57 (0.46–0.73)	<0.001
1–2 child [ref: no children]	0.99 (0.63–1.55)	0.953		
⩾3 children [ref: no children]	1.06 (0.54–2.09)	0.870		
Planned pregnancy	0.94 (0.57–1.53)	0.795		
Unemployed [ref: employed]	1.16 (0.73–1.85)	0.534		
Student [ref: employed]	0.97 (0.38–2.47)	0.957		
Financial support from partner	0.74 (0.45–1.21)	0.236		
Child support grant	1.01 (0.64–1.61)	0.943		
Any social support grant	0.97 (0.61–1.55)	0.911		
People/sleeping room	0.89 (0.72–1.08)	0.239		
HIV-infected	1.09 (0.62–1.93)	0.762		
⩽6 months post birth [ref: pregnant]	0.90 (0.55–1.45)	0.656		
Miscarriage/stillbirth [ref: pregnant]	2.41 (0.74–7.86)	0.145		
Probable CMD^a^	1.69 (0.97–2.93)	0.061	1.35 (1.02–1.79)	0.038

a Common mental disorder, ⩾13 on the Edinburgh Postnatal Depression Scale (EPDS).

## Discussion

We used data collected at two time-points, 3 months apart, during the COVID-19 pandemic in Cape Town, South Africa to identify social, economic and mental health-related variables associated with food insecurity, probable CMD and domestic violence in perinatal women attending public healthcare facilities situated in low socio-economic communities. At baseline, we found that 80% of perinatal women lived in food insecure households, 21% experienced domestic violence and 12% had probable CMDs. Three months later, the prevalence of food insecurity, domestic violence and probable CMD was significantly lower. In cross-sectional analysis at both baseline and follow-up, we found that food insecurity was significantly associated with race, several markers of multidimensional poverty (including employment status, receipt of grants and number of people per sleeping room), probable CMD, psychological distress and experiences of domestic violence; while probable CMD was significantly associated with experiencing psychological distress, food insecurity and domestic violence. These cross-sectional associations reflect a clustering of socio-economic adversity and worse mental health, which has been observed in previous perinatal studies (Abrahams *et al*., 2018; Malan *et al*., 2018). Using multi-level multivariable regression modelling, we found that being unemployed and having a probable CMD at baseline significantly increased the risk of being food insecure at follow-up. We were also able to show the bidirectional relationship between domestic violence and probable CMD, using multi-level multivariable regression analysis.

Following the strict lockdown imposed by the South African government in March 2020, the World Health Organisation praised South Africa for its effective fight against COVID-19 (eNCA, 2020). However, the same lockdown measures used to curb the spread of the COVID-19 pandemic disproportionately affected those living in low-resource settings (Laborde *et al*., 2020). In South Africa, where 29% of the population were already unemployed (Statistics South Africa, 2019*b*) and 35% of employed individuals worked in the informal sector, the lockdown plunged millions more into unemployment, poverty and food insecurity (Rogan and Skinner, 2020). In June 2020, when the lockdown restrictions had just started easing, we found that 54% of perinatal women in our baseline sample were unemployed and 80% were living in food insecure households. Three months later, we found that the number of unemployed had risen by 16%, yet the proportion of women living in food insecure households had decreased by 11%. The decrease in the proportion of food insecure households is likely due to measures the South African government employed, such as providing unemployed individuals who were over 18 years with a COVID-19 social relief and distress grant, valued at R350 (US$25) per month (Baskaran *et al*., 2020). In addition, the South African government (Western Cape Government, 2021), as well as several non-profit organisations, distributed thousands of food parcels and food vouchers to those in need (DG Murray Trust, 2021; Gift of the Givers Foundation, 2021; Matlwa Mabaso *et al*., 2021; Siyabonga Africa, 2021).

Even though the levels of food insecurity were significantly lower at follow-up, more than two-thirds of the perinatal women were still living in food insecure households. In South Africa, persistent food insecurity is associated with the double burden of malnutrition, where undernutrition co-exists with overweight and obesity in the same household and is characterised by an overweight or obese mother living with her underweight, wasted or stunted child (Popkin *et al*., 2020). Pregnant and lactating women, with their increased nutrient requirements, are particularly vulnerable to malnutrition (Desyibelew and Dadi, 2019), which can take the form of underweight or obesity. Malnutrition is associated with anaemia, hypertension and haemorrhage in the mother and pre-term delivery, low birth weight and intrauterine growth retardation in infants (Papathakis *et al*., 2016).

We found that both the prevalence of probable CMD and experiences of psychological distress decreased between baseline and follow-up. Similar prevalence estimates have been reported in studies on postnatal CMDs from Ethiopia (Dadi *et al*., 2020) and Zimbabwe (January *et al*., 2017) where the prevalence ranged between 9% and 34%. The low prevalence of probable CMD at baseline and follow-up is likely because the majority of women were in the third trimester of pregnancy or had already given birth, as studies have found that mild to moderate symptoms of depression improve during the last trimester of pregnancy and after birth (Christensen *et al*., 2011; Garman *et al*., 2019), and that the prevalence of depression is higher during pregnancy compared to after childbirth (Underwood *et al*., 2016).

Several systematic reviews have described the impact of food insecurity on poor mental health. The studies included in the reviews provide evidence that food insecurity increases the risk of CMDs throughout the life cycle (Weaver and Hadley, 2009; Jones, 2017; Pourmotabbed *et al*., 2020; Trudell *et al*., 2021). Qualitative studies report that food insecurity is experienced as acute psychological suffering, with overwhelming feelings of shame (Weaver and Hadley, 2009). These findings are in keeping with the social causation pathway which hypothesises that unfavourable social and economic conditions such as poverty, food insecurity and domestic violence increases the risk of poor mental health (Lund and Cois, 2018). While we found that food insecurity was significantly associated with probable CMD at baseline and follow-up, in our multivariable model, being food insecure at baseline did not significantly increase the odds of probable CMD at follow-up. Instead, our findings reflect a complex picture in which some socio-economic risk factors experienced at baseline, particularly domestic violence, predict probable CMD prevalence at follow-up; and probable CMD prevalence experienced at baseline predicts worse socio-economic outcomes such as food insecurity and domestic violence at follow-up. Our study reported lower levels of domestic violence than other studies in similar settings (Malan *et al*., 2018; Schneider *et al*., 2018) – even though South Africa experienced a surge in domestic violence during the COVID lockdown (Nduna and Tshona, 2021). The lower levels may be due to the telephonic data collection method used as the women being interviewed may not have had sufficient privacy to disclose their experiences of domestic violence. Yet, in keeping with the finding of Malan *et al*. (2018) and Schneider *et al*. (2018), and in support of the social causation hypothesis, we show that experiencing domestic violence predicts probable CMD. Furthermore, our study provides evidence of a bidirectional relationship, whereby domestic violence experienced at baseline predicts probable CMD, and probable CMD experienced at baseline predicts domestic violence.

While many studies have examined the social causation pathway, few studies investigated the impact of poor maternal mental health on food insecurity. A study in Cape Town reported that the odds of household food insecurity were four times greater in pregnant women who were depressed (Abrahams *et al*., 2018), and a birth cohort study, located in a small city outside Cape Town, reported that maternal depression in a predominantly Black African community significantly predicted perceived household food insecurity (OR 1.08; *p* < 0.01) (Pellowski *et al*., 2017). Our study found that having probable CMD increased the subsequent odds of household food insecurity. However, we may not have enough evidence to support the social drift hypothesis which proposes that people living with a mental illness drift into poverty due to the stigma of mental illness, increased spending on healthcare and reduced income-generating potential associated with the disability of their mental illness (Lund and Cois, 2018). We did not measure mental health stigma, and our data were collected at facilities where healthcare was available at no cost. In addition, the 3-month follow-up period and the low prevalence of depression may mean that the increased level of unemployment is a consequence of the COVID-19 lockdown (Rogan and Skinner, 2020), and not necessarily a result of poor mental health.

Our study has strengths and limitations. We interviewed women attending 14 randomly selected MOUs and BANC clinics in low-resource settings in Cape Town. This allowed us to generalise our findings to low-resource settings in Cape Town, instead of being limited to a smaller population. Interviewer bias may be present as 14 fieldworkers were telephonically trained to administer the questionnaires and we were not able to assess inter-rater reliability. A second limitation was the low follow-up rate, with one-quarter of baseline participants lost to follow-up. However, they were not significantly different to those who were not lost to follow-up with regards to several socio-demographic characteristics measured at baseline. Response bias may be responsible for the unusually low prevalence of probable CMD and domestic violence observed at both baseline and follow-up, as fieldworkers may not have built up enough rapport with the participants to ensure full disclosure of sensitive information; or study participants may have not been able to find a private space in which to conduct the telephonic interview. We did not have a control group, which would have allowed us to better understand the effect of the COVID-19 lockdown on the women's mental health and experiences of food insecurity. We did not do any qualitative interviews which would have helped us understand the relationship between poor mental health and food insecurity. Our follow-up time period was relatively short, limiting any causal inference from the longitudinal analysis.

Further research is needed to better understand how experiences of mental health problems lead to food insecurity and malnutrition among pregnant and lactating women, as well as to investigate the long-term effects of food insecurity on mothers and infants born during the pandemic.

## Conclusions

This study provides evidence that perinatal women with poor mental health and adverse social and economic challenges are at an increased risk of food insecurity and domestic violence, and conversely that domestic violence increases the risk of later CMD. While the lockdown restrictions imposed during the COVID-19 pandemic may have decreased COVID-related illness and death, it came at a high price for those living in already vulnerable circumstances. The effect of the high levels of food insecurity and related poor mental health of pregnant and lactating women during the COVID-19 pandemic indicates the need for interventions to address mental health, food insecurity and domestic violence among pregnant women in low-resource settings.

## Data

The datasets used and/or analysed during the current study are available from the corresponding author on reasonable request.
